# A Review of the Most Commonly Used Methods for Sample Collection in Environmental Surveillance of Poliovirus

**DOI:** 10.1093/cid/ciy638

**Published:** 2018-10-30

**Authors:** Graciela Matrajt, Brienna Naughton, Ananda S Bandyopadhyay, John Scott Meschke

**Affiliations:** 1 Department of Environmental and Occupational Health Sciences, School of Public Health, University of Washington, Washington; 2 Bill & Melinda Gates Foundation, Seattle, Washington

**Keywords:** poliovirus, environmental surveillance, sampling

## Abstract

We performed a review of the environmental surveillance methods commonly used to collect and concentrate poliovirus (PV) from water samples. We compared the sampling approaches (trap vs grab), the process methods (precipitation vs filtration), and the various tools and chemical reagents used to separate PV from other viruses and pathogens in water samples (microporous glass, pads, polyethylene glycol [PEG]/dextran, PEG/sodium chloride, NanoCeram/ViroCap, and ester membranes). The advantages and disadvantages of each method are considered, and the geographical areas where they are currently used are discussed. Several methods have demonstrated the ability to concentrate and recover PVs from environmental samples. The details of the particular sampling conditions and locations should be considered carefully in method selection.

Poliovirus (PV) is a human enterovirus that is the causative agent of paralytic poliomyelitis [1, 2]. There are 3 strains of wild PV (WPV1, WPV2, and WPV3). WPV2 was declared eradicated by the World Health Organization (WHO) on 20 September 2015 [3], and WPV3 has not been detected anywhere in the world since November 2012 [4]. With effective programmatic use and delivery of vaccines, the Global Polio Eradication Initiative (GPEI) has made significant advances toward the eradication of PV, and since 1988, overall polio cases have decreased by more than 99% [5]. Transmission of WPV has not yet been interrupted in Afghanistan, Pakistan, and Nigeria [6]; however, reintroduction of WPV poliomyelitis in many previously polio-free countries is a real threat [7].

There are 2 types of vaccines to stop polio transmission: inactivated polio vaccine (IPV) and oral polio vaccine (OPV), which consists of a mixture of live attenuated PV strains of each of the 3 serotypes known as Sabin strains 1, 2, and 3 [8, 9]. Although successful use of OPVs has driven the virus out of most countries in the world, the live attenuated vaccine can revert to neurovirulence and get transmitted from person to person as a circulating vaccine-derived PV strain (VDPV) in settings of low immunization coverage [10].

VDPVs, derived from each of the 3 OPV serotypes, are a mutated version (1% to 15% difference in sequence homology from the Sabin strains) of the attenuated Sabin-like virus used for oral vaccination [8, 11–13]. There are 3 categories of VDPVs: immunodeficient (iVDPVs), circulating (cVDPVs), and ambiguous (aVDPVs). iVDPVs arise from prolonged replication of VDPVs in individuals with rare immune deficiency disorders; cVDPVs arise when the virus circulates for a long period of time in a community and thus have evidence of person-to-person transmissibility and neurovirulence; and aVDPVs represent a category of virulent PVs that cannot easily be assigned to iVDPV or cVDPV, such as some environmental isolates [14].

cVDPVs have caused several outbreaks in different parts of the world and remain a key risk to the polio endgame [15–18]. Since 2000, 95% of the cVDPVs have been derived from the type 2 strain of OPV [19]. With the worldwide switch in April 2016 from trivalent to bivalent OPV, which does not contain the type 2 strain, the risk of cVDPV2 has been reduced [19, 20]. However, cVDPV2 outbreaks still occur, particularly in conflict zones, such as in Syria and Democratic Republic of Congo where there are ongoing cVDPV2 outbreaks [21].

PV surveillance is a crucial component of the GPEI endgame strategy and plays an important role in detecting ﬁnal reservoirs of WPV and VDPVs. The 2 primary aspects of PV surveillance include acute ﬂaccid paralysis surveillance (AFPS) and environmental surveillance (ES). The gold standard for PV surveillance is based on investigations of the cause of all cases of AFP in children aged ≤15 years to determine whether the AFP was caused by PV [22]. Because infected individuals excrete PV in feces for periods of up to several weeks regardless of symptoms, ES includes the analysis of sewage and other wastewaters (fecally impacted and sewage-impacted waters) to determine whether PVs are present in samples collected from these sources [7]. ES has historically been considered a supplementary approach to AFPS. However, it is resource intensive to maintain a wide and sensitive AFPS system for the long term as such a system relies heavily on trained medical professionals for clinical and neurological evaluation and reporting of cases, in addition to its dependence on field- and laboratory-based activities. Also, silent circulation of PV in the environment is a serious concern [12], which is why ES has become increasingly important. ES is especially crucial for people living in high-risk regions, such as underimmunized populations at risk of WPV or VDPV transmission or importation [23, 24].

Though a crucial aspect of the endgame strategy, there are significant challenges with ES, including considerations of key attributes of the geographical areas, catchment populations (size and location), type and characteristics of sewage and treatment systems, and available laboratory resources [25]. Additionally, PV needs to be concentrated from wastewater samples before it can be detected and characterized, and most methods and tools developed over the past decades are only operational for small (tens of milliliters) quantities of water, waters with no turbidity, and low flow rates. PV, as with other enteric viruses, is relatively difficult to concentrate from wastewaters due to its low occurrence and small size [26]. The methodology for ES includes the collection of wastewater samples from a variety of sources and transportation to a laboratory where the samples are processed. The samples are concentrated to reduce the eluate volume to a minimum. Then, the eluate is analyzed by tissue culture to determine the presence of viruses. Finally, the viruses are characterized by polymerase chain reaction (PCR), serotyping by intratypic differentiation, and sequencing to determine the type and strain present in the sample.

## TOOLS CURRENTLY AVAILABLE

Currently, 2 sampling methods are used in the field to collect water samples: “trap” and “grab” (Table 1). Among the grab methods, 2 process methods are used to concentrate the volume in order to optimally screen for the presence of PV: precipitation and filtration. The WHO guidelines for PV ES recommend grab sampling of 500 mL of wastewater with a 2-phase separation method [8, 27]. As the application of these methods to wastewater is crucial for the detection of PV, the purpose of this review is to describe the current methods for collecting and concentrating wastewater samples for PV during ES processes. As the polio endgame nears, risks of WPV reemergence and VPDV circulation will increasingly need to be mitigated with an efficient and sensitive ES system that is able to detect the pathogen silently circulating in the environment before large outbreaks can occur. This review highlights the key strengths and limitations of the PV detection methods in use that can help inform programmatic choices for the endgame and beyond.

**Table 1.** Methods for Virus Acquisition and Concentration From Water Samples

Sampling Method	Process Method	Tools/Chemicals	Advantages	Disadvantages	Countries Where They Are Used	References
Trap sampling		Gauze/pads	Simple, effective, inexpensive for large volumes of water	Not quantitative	Brazil, Canada, Colombia	[15, 29, 31]
		Macroporous glass	Effective for large volumes of water	Not quantitative	…	[8]
Grab sampling	Precipitation	One point/composite	Quantitative method; effective for collecting several samples at different times of day; automated system allows collection during peak hours of flow	…	Israel, Brazil, Finland	[8, 13]
		PEG/dextran (2-phase)	World Health Organization recommended method; straightforward, does not require complex equipment or reagents	Only effective for small volumes (500 mL); sample needs to be transported to a laboratory, samples cannot be processed in situ	Taiwan, Nigeria, Italy, India, Indonesia, Iran, Pakistan, Mexico	[8, 13, 17, 18, 33–42]
		PEG/sodium chloride	Works for the identification of several types of enteric viruses; simple; requires little processing time and modest skills	Works only with small volumes; requires a centrifugation step that might raise costs	Israel, India, Kenya, South Africa, Hispaniola, United States	[12, 43–52]
	Filtration	NanoCeram/ViroCap	Membrane is positively charged, water preconditioning is not needed; operates over a wide range of pH; field deployable; works with large volumes (>1000 L); commercially available; Small elution volume (larger concentration factor); easy to use; high filtration rates; recommended by the US Environmental Protection Agency; able to adsorb poliovirus 1 over a broad pH range; inexpensive	Filter clogging when used in highly turbid waters	France, Cambodia, United States	[56, 64, 74, 76, 77]
		Mixed ester cellulose membrane	Commercially available; inexpensive	Membrane is negatively charged; water needs to be centrifuged first; preconditioning with MgCl_2_ to adjust pH is required prior to filtration, so not field deployable; works with small (0.5–1 L) volumes	United States, Japan, China	[30, 54, 56, 59, 79–83]

Abbreviation: PEG, polyethylene glycol.

### Trap Method

In the trap method, a bag of nonspecific absorbing material is hung in the sewage stream. After several days, the bag is removed and shipped to the laboratory, where the absorbed material is eluted and analyzed for the presence of PVs [8]. Common adsorbing materials are gauze pads and other cotton-made fabrics, as well as macroporous glass in permeable bags [8].

Trap sampling methods are relatively simple, and the devices used are inexpensive and effective for sampling large volumes of wastewater. However, these methods are only qualitative since it is difficult to determine the total volume of water that passes through the pads/glass. Consequently, their sensitivity and recovery efficiency are poorly defined.

#### Moore Swab

This method of trap sampling is based on the absorption capabilities of tissue, gauze, and pads when submerged in sample water for several hours [28]. The pads are made of cotton, which is very absorbent, and are kept in contact with the sample water for at least 48 hours. To increase surface contact, the pads are folded several times before being inserted into a socket. The socket is then suspended with strings or chains into the wastewater stream [29]. The pads are sent inside sterile bags to be processed in the laboratory by organic flocculation and chloroform clarification [15], by filtration with mixed cellulose ester membranes [30], or by soaking in 3% beef extract [31]. This method was extensively used to analyze wastewater for waterborne viruses prior to development of the 2-phase concentration method [8, 13] and was very successful at detecting WPV, Sabin-like strains, non-Sabin–like strains [29, 31], and vaccine-derived strains [15].

#### Etched/Macroporous Glass

This method uses macroporous glass inside permeable bags [8]. In the field, a sorbent bag with sorbent glass is fixed using fishing line so that the bag hangs in the wastewater stream. After exposure for 3–7 days, the sorbent bag is placed in a separate plastic parcel or sterile flask and transported to the laboratory in a cold bag or cold box for further analysis. To release PV trapped in the ground glass–containing bags, the glass powder is first transferred to a small glass column. The bag with sorbent is placed in a sterile Petri dish. The edge of the sorbent bag is cut off, and the glass sorbent is washed out with sterile distilled water (approximately 5 mL) using a pipette. The glass is then poured into a column of 5–10 mL volume. The column is sequentially rinsed with defined buffer solutions. Viruses are eluted stepwise with 3 sterile solutions, and the eluates are treated with chloroform and subsequently analyzed. Glass columns are pretreated by wetting the column (inner surface) with silicone fluid to prevent unwanted adsorption of viruses to the column wall.

### Grab Method

In the grab method, an amount of raw sewage is collected at a selected sampling site, either at 1 point in time or at predetermined times to form a time-adjusted composite sample. Many sewage treatment plants use automated equipment to collect samples at regular intervals during a 24-hour period or during the peak hours of household sewage flow. The larger the volume of sewage analyzed, the higher the theoretical sensitivity to detect PV circulation in the source population. However, volumes larger than 1 L can be difficult to handle in the laboratory and may be replaced by several parallel regular samples [8].

Grab sampling is generally preferred to trap sampling because it is a more quantitative method that allows estimation of the system’s detection sensitivity [8]. Moreover, long-term experience suggests that programs that use concentrated grab samples detect PVs and non-polio enteroviruses more often than those that use trap samples [8]. Currently, grab sampling is the collection method used and recommended by the GPLN/WHO to obtain samples for polio ES [13].

### Precipitation Methods

Precipitation methods are used to concentrate viruses from the ES wastewater samples. These methods can be used as a primary concentration method when the sample is first processed or they can be used as a secondary concentration method when the viruses are recovered (eluted) from the filter/membrane.

#### Polyethylene Glycol and Dextran

This method is based on the differential weight between different particles in a water sample and how they distribute and separate between 2 distinct phases [32]. Polyethylene glycol (PEG) is a polymer that can be combined with another polymer/molecule of different weight, typically dextran. The mixture interacts with the water sample and, after intense shaking, separates on the basis of their 2 weights, carrying along viruses that have similar weight. Viruses can be recovered from the denser phase for further characterization. The PEG/dextran method is the WHO-recommended concentration/separation method (referred to as the 2-phase separation method [8, 13]) for ES of PV. A main strength of this method is that it is relatively straightforward and does not require complex reagents or equipment. The volume of water sample to be concentrated is 500 mL and, after concentration, the volume of the obtained eluate is 10–15 mL, resulting in a minimum effective volume assayed of 150 mL [13]. The resulting nominal sample concentration is approximately 50-fold.

The 2-phase separation method has been extensively used during PV ES campaigns and has been very successful at detecting WPV [32–36], Sabin-like PV strains [17, 34, 36–42], VDPV [17, 18, 41], and other PV strains [35].

The 2-phase method has some limitations. It is only effective for small volumes (0.5–1 L), which impacts sensitivity. Moreover, although sampling is done in the field, sample processing to separate the virus from the water sample needs to be done in a laboratory [27].

#### PEG and Sodium Chloride

PEG combined with sodium chloride (NaCl) has been used as an alternative precipitation method, often as a secondary concentration method, or combined with other concentration methods for the detection of PVs. PEG combined with NaCl forms flocs that yield virus-containing sediment. This method has been used to identify Sabin-like and cVDPV strains [43–47], WPV strains [43, 48], and WPV1 South Asia (SOAS) PV strains [12, 49, 50]. It has also been used to reconcentrate other enteric viruses from wastewater samples [51, 52].

PEG/NaCl has been used for ES in several locations including Ecuador, where 1–3 L of both sewage and stream water were collected in areas of poor vaccination coverage and high population density [44]. Sabin-like strains were found in the ES samples, and the method was useful in identifying deficiencies in vaccination coverage and AFPS. However, the methodology’s sensitivity is limited since the study failed to find all AFP-reported cases [44]. In South Africa, 50 mL of sewage and river water (used for washing purposes) samples were collected, and all 3 serotypes of Sabin-like strains were found (49 isolates) [46]. In Hispaniola (Haiti and Dominican Republic), where 1-L sewage, latrine, and stream water samples were collected near villages where clinical PV cases had been reported and where vaccine coverage was low, all 3 serotypes of cVDPV were identified (in 95% of samples collected). In India, multiple studies have collected 1- to 2-L sewage samples in areas endemic for poliomyelitis and were identified as having poor sanitation and being densely populated. Both Sabin-like (all 3 serotypes) and WPV1 were identified (137 isolates) in one study [43], while in an earlier study WPV1 (35 isolates) and WPV3 (1 isolate) were identified [45]. In Israel, 10 L of 24 hour-composite sewage samples (0.5- to 1-L individual samples) were collected in areas where the WVP1-SOAS strain had been detected and circulated with high incidence (20 isolates) [12, 49, 50].

This method works best with wastewater sample volumes that range from 50 mL to 3 L [43, 44, 46–50]. This is a fairly simple method that requires modest skills and little processing time, but it does require a centrifugation step that might raise the processing costs [47].

### Filtration Methods Based on Charge

Filtration methods are used to concentrate viruses and are based on the principle that viruses can be adsorbed to or retained in a filter medium and then be eluted with an organic solution. These methods are based on charge adsorption and are widely used for PV and other enteric viruses [53]. The filters come in 2 different formats: flat disk/membrane [26, 31, 54–60] or packed in a cartridge [61–63]. The filters have a variety of compositions and flow rates that will determine the charge, particle size, and amount of sample that can be filtered.

PV, like most enteric viruses, has a slight negative charge at neutral pH [64]. As a result, positively charged filter media is used to facilitate the adsorption of PV to the filter; a change in pH modifies the virus’s natural charge (or that of the filter media) to facilitate elution of the virus from the filter. Electrostatic forces are instrumental in virus–filter interactions. For example, strains of PV1 have a net positive surface charge if the pH of the surrounding medium remains below the main virus isoelectric point (pI, approximately 6.5–7.1). However, when the medium pH surpasses the pI, the virus acquires a negative net surface charge [65]. Charge-based methods use these properties to adsorb PVs on media that are either positively or negatively charged. For negative-charge filters, it is often necessary to acidify the water samples and to add multivalent cation salts magnesium chloride (MgCl_2_) or aluminum chloride (AlCl_3_) in order to facilitate and optimize virus adsorption to filter surfaces [64, 66].

### Electropositive-charged Filters

The NanoCeram filter (Argonide, Sanford, FL) is a commercially available electropositive filter. The US Environmental Protection Agency recommends this method for virus monitoring in environmental and drinking waters [67]. The filter comes in a cartridge or flat disc format. The cartridge can be purchased alone or as an encapsulated filter under the name ViroCap (Scientific Methods, Inc., Granger, IN).

NanoCeram is a nonwoven, pleated, microporous filter made of a multilayer of microglass filaments coated with highly electropositive nanoalumina (AlOOH) fibers (approximately 2 nm in diameter by approximately 250 nm in length) derived from the mineral boehmite [68, 69]. These fibers are dispersed throughout a cellulose and polyester fiber matrix with a 2 μm average pore size [68, 70, 71], giving the filter an extensive surface area (approximately 500 m^2^/g), a high isoelectric point, and a strong electropositivity [69].

A strength of NanoCeram filters is that they are inexpensive [69] and efficiently adsorb PV over a broad pH range (6.0–9.5) [70]. These filters work well with wastewater sample volumes of 10–40 L [72–76].

NanoCeram filters have been validated in the field for the recovery of PV in several locations. In the United States, 10-L seeded (PV1, PV2, and PV3) effluent wastewater samples were filtered, and PVs were recovered with a 50%–57% efficiency [72] and PV1 was recovered with a 33%–39% efficiency [74]. Also in the United States, 40 L of treated wastewater spiked with PV1 was filtered and the virus recovery was 57% [76].

ViroCap filters are disposable capsule cartridges filled with the same material that is inside the NanoCeram filters, that is, nanoalumina fibers (boehmite) infused into a microglass/cellulose matrix with an average pore size of 2–3 µm [70, 77]. These filters are economical, easy to use, and field deployable [78].

ViroCap filters have been validated in the field in several locations. In the United States, 10-L seeded (PV1) influent wastewater samples were filtered, and PV1 was recovered with a 69% efficiency [73, 74]. In Kenya, 3- to 4-L wastewater and wastewater-impacted surface water samples were filtered using ViroCap filters within a bag mediated filter system [74].

### Electronegative-charged Filters

Negatively charged mixed cellulose ester membranes have a 0.45 μm pore size and are commercially available (Advantec, Millipore). This membrane has been successfully used to detect various strains of PV in sewage and river-contaminated water samples [30, 54, 56, 59, 79–83].

One disadvantage of this type of filter is that a centrifugation step is always required before filtration through the membrane [30, 54, 56, 59, 79–83]. Consequently, it is not field deployable. Another limitation is that it is necessary to precondition the water samples to pH 5 or lower with the addition of MgCl_2_ in order to achieve optimal virus detection when molecular methods are used, in particular, PCR amplification [30, 54, 56, 59, 79–82]. A modification of this method involves use of AlCl_3_ to treat the water sample prior to being filtered in order to form a cation-coated filter [84].

This membrane filter has been validated in several locations. In Japan, approximately 1 L of sewage water samples collected from wastewater treatment plants were filtered, and Sabin strains of the 3 serotypes were detected in 72 isolates [79], 83 isolates [56], and 31 isolates [54]. In China, 0.5-L wastewater samples collected from a wastewater treatment plant were filtered, and a Sabin-like recombinant type 2/3 PV was detected [30]. Also in China, 0.5–1 L of sewage samples collected from treatment plants were filtered, and Sabin-like PV types 1, 2, and 3 were detected in 18 isolates [80], 39 isolates [81], 32 isolates [82], and 168 isolates [83], and VDPV type 2 was detected in 1 isolate [59, 83].

## DISCUSSION

Here, we describe the methods currently used to collect and concentrate wastewater samples for PV ES. Strengths and limitations are highlighted, which can help inform programmatic choices.

Trap sampling methods are simple, inexpensive, and effective at sampling large volumes of wastewater but are limited to qualitative analysis instead of a more robust quantitative assessment. As a result, the sensitivity and recovery efficiency are poorly defined. At present, these methods are generally not preferred and rarely used compared to grab sampling methods. Grab sampling is more quantitative and allows an estimation of detection sensitivity. The WHO has recommended the grab sampling approach for obtaining samples for PV ES.

Precipitation methods are frequently used to concentrate viruses from small-volume wastewater samples and include methods such as PEG/dextran (otherwise known as the 2-phase separation method) and PEG/NaCl. Although the PEG/NaCl method has been used effectively for PV ES, the process requires more advanced laboratory technology (such as centrifugation) than PEG/dextran, which is the WHO-recommended concentration/separation method and has been extensively and effectively used for PV ES.

Charge-based methods involve the use of positively charged filters such as NanoCeram and ViroCap filters or negatively charged membranes such as mixed cellulose ester membranes. Although membrane filters have been successfully used to detect various strains of PV in sewage and river-contaminated water samples, these methods can be limited by filter loading in samples that contain heavy solids and by the need for pumps or vacuum systems. The NanoCeram/ViroCap method has been validated in field studies and adapted to concentrate samples in the field.

The methods discussed here allow concentration of PV from ES samples. Each presents some technical limitations such as the need for a centrifugation step, the need to precondition the water, a poor sensitivity with turbid waters, or the need for large sample volumes. In addition to these limitations, the choice of one method over another may depend on safety and security issues in the field, which are highly dependent on the sampling location. Extreme temperatures (up to 40°C–50°C) at some locations may complicate the sampling process. Sampling staff who are exposed to very high temperatures for long periods of time while in the field may experience health impacts. Further, elevated temperatures may complicate reverse cold chain transport of samples.

The economic aspects of the different ES methods discussed here were outside the scope of this review, as were the geographical limitations of ES and the percentage of areas covered by ES. This information is provided elsewhere [25]. Here, we focused on the feasibility of field deployment of the existing methods. We found that several methods may be effectively deployed for ES and safely used in the field. This review suggests that it would be beneficial to develop explicit performance standards and proficiency testing panels to validate the methods selected for ES programs in different regions.

## CONCLUSIONS

As the extent of WPV circulation continues to decline, there is a growing need to maintain and enhance the polio surveillance network and its sensitivity to ensure no area of active PV circulation is missed. Additionally, with major and unprecedented changes in immunization schedules in recent times, such as the withdrawal of trivalent OPV and switch to bivalent OPV for polio protection, timely identification of any Sabin 2 or vaccine-derived circulation from type 2 have become program priorities. Compared to AFPS, ES for polio is an inexpensive method that can be deployed in the field with relatively fewer highly trained staff. Also, it is considered highly sensitive and as the infection to AFP ratio declines with the increasing use of IPV and planned, successive withdrawal of different types of OPV, expanded deployment of ES should ensure higher likelihood of detecting the silent circulation of PV. Considering the critical and growing importance of ES in the current phase of the polio eradication endgame, continuous evaluation of the different sampling tools and methods and context-specific applications are key to the program’s success. “One size” may not “fit all” as the virus load in the environment continues to decline, local challenges predominate in smaller geographic areas, and the economic dimensions of maintaining robust AFPS for the long term become more apparent. Adapting current methods and adopting new tools for polio ES may be critical as the epidemiology of different types of PV evolve with changing vaccine choices. The findings presented here should inform policy makers about the spectrum of available choices for polio ES and encourage possible new innovations to shape the future of polio ES, ensuring global readiness to detect and respond to PVs both now, as we are on the verge of eradication, and long after eradication is achieved.

## Notes

***Financial support*.** This work was funded through a subaward from PATH (GAT.01558814-SUB).

***Supplement sponsorship.*** This article appears as part of the supplement “Polio endgame and beyond: Vaccine choices, transmission dynamics, and surveillance implications,” sponsored by The Bill & Melinda Gates Foundation.

***Potential conflicts of interest.*** All authors: No reported conflicts of interest. All authors have submitted the ICMJE Form for Disclosure of Potential Conflicts of Interest. Conflicts that the editors consider relevant to the content of the manuscript have been disclosed.
